# Response to "Neglecting normalization impact in semi-synthetic RNA-seq data simulation generates artificial false positives" and "Winsorization greatly reduces false positives by popular differential expression methods when analyzing human population samples"

**DOI:** 10.1186/s13059-024-03232-8

**Published:** 2024-10-30

**Authors:** Xinzhou Ge, Yumei Li, Wei Li, Jingyi Jessica Li

**Affiliations:** 1 Department of Statistics and Data Science, University of California, Los Angeles, CA 90095 USA; 2 Present Address: Department of Statistics, Oregon State University, Corvallis, OR 97331 USA; 3 Division of Computational Biomedicine, Department of Biological Chemistry, School of Medicine, University of California, Irvine, CA 92697 USA; 4 Present Address: School of Biology and Basic Medical Sciences, Soochow University, Suzhou, 215123 China; 5 Interdepartmental Program in Bioinformatics, University of California, Los Angeles, CA 90095 USA; 6 Department of Human Genetics, University of California, Los Angeles, CA 90095 USA; 7 Department of Computational Medicine, University of California, Los Angeles, CA 90095 USA; 8 Department of Biostatistics, University of California, Los Angeles, CA 90095 USA

## Abstract

Two correspondences raised concerns or comments about our analyses regarding exaggerated false positives found by differential expression (DE) methods. Here, we discuss the points they raise and explain why we agree or disagree with these points. We add new analysis to confirm that the Wilcoxon rank-sum test remains the most robust method compared to the other five DE methods (DESeq2, edgeR, limma-voom, dearseq, and NOISeq) in two-condition DE analyses after considering normalization and winsorization, the data preprocessing steps discussed in the two correspondences.

**Supplementary Information:**

The online version contains supplementary material available at 10.1186/s13059-024-03232-8.

## Introduction

As correspondences to our published study [1], Hejblum et al. [2] and Yang et al. [3] have raised issues or comments about our analyses. Here, we respond to the two correspondences and show that they do not challenge the major finding of our study—DESeq2 and edgeR have exaggerated false discoveries when analyzing human population samples (Fig. 1 in [1])—which is, in fact, exempt from the issues raised in Hejblum et al. [2].

**Fig. 1:**
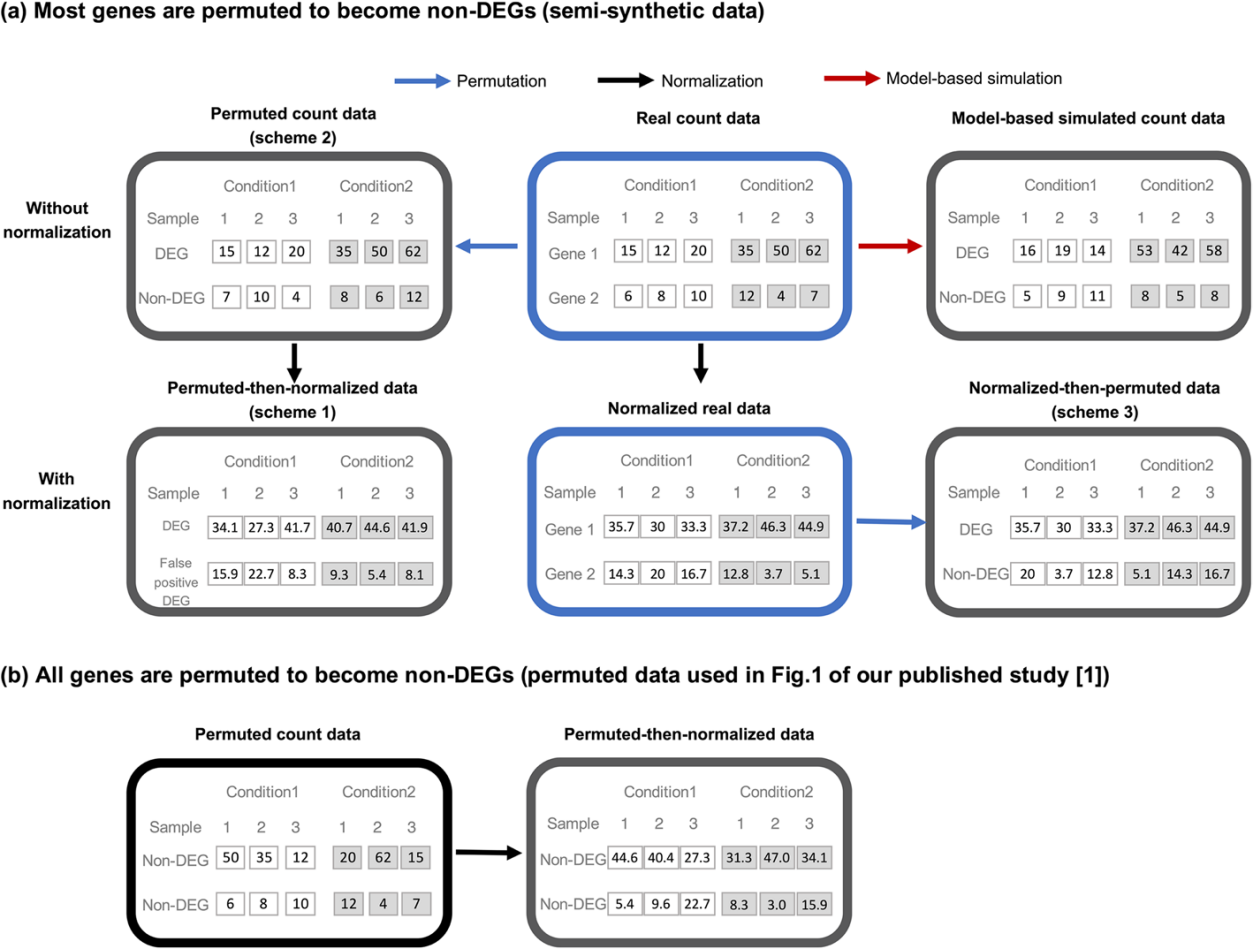
Illustration of **a** the two strategies for generating semi-synthetic data from real data and **b** the generation of permuted data used in Fig. 1 of our published study [1]. **a** Permutation-based strategy and model-based strategy for generating semi-synthetic data. There are three schemes for the permutation-based strategy: scheme 1—“permutation first” generates semi-synthetic data by permuting the real count data, followed by normalization and then DE analysis (bottom-left); scheme 2—“no normalization” generates semi-synthetic data by permuting the real count data, followed by DE analysis directly, without normalization (top-left); scheme 3—“normalization first” generates semi-synthetic data by normalizing the real data (bottom-middle), which are no longer counts, followed by permutation and then DE analysis (bottom-right). In the model-based strategy (top-right), we fit a multi-gene NB distribution to the real samples using the simulator scDesign3 [4]. For each true differentially expressed gene (DEG) we fit a NB distribution using the samples under each condition; for each true non-DEG, we fit one NB distribution by pooling the samples from both conditions; then, we generate semi-synthetic data by sampling from the fitted multi-gene NB distribution. **b** For the analysis in Fig. 1 of our published study [1], all genes are permuted and become true non-DEGs, followed by normalization and then DE analysis

**Fig. 2:**
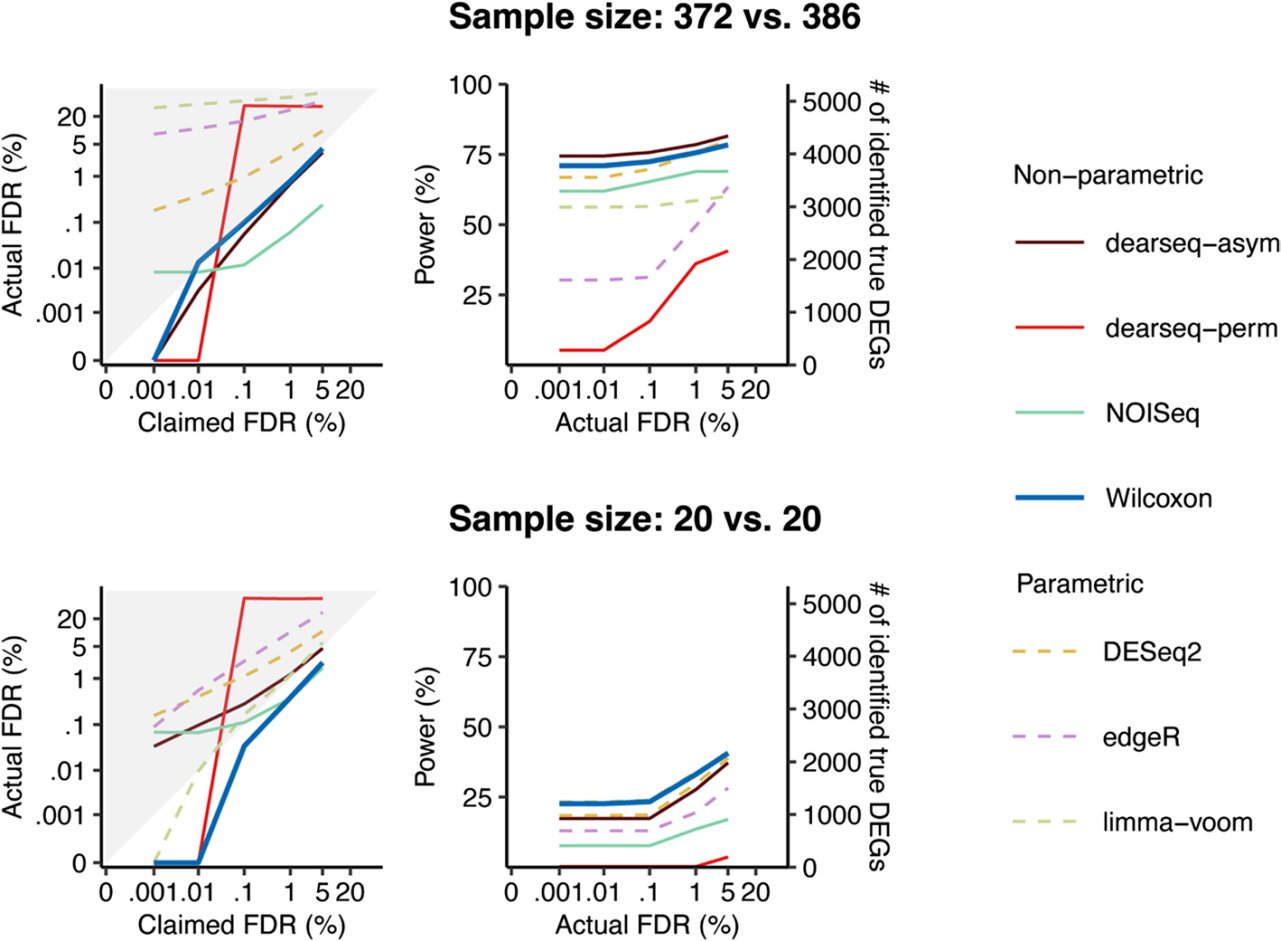
Comparison of DE methods on semi-synthetic data generated using the permutation-based strategy from GTEx data of heart left ventricle vs. atrial appendage under the data generation scheme 2. The FDR control (left panel) and power given the actual FDRs (right panel) under a range of FDR thresholds (i.e., claimed FDRs) from 0.001 to 5% for 2 sample sizes: all samples from the 2 conditions (top panel) and 20 samples per condition (bottom panel)

There are two major points in Hejblum et al. [2]. First, in the power comparison results (Fig. 2 in our published study [1]), the semi-synthetic data did not have all genes permuted as true non-DEGs because we kept the unpermuted genes as true DEGs for power calculation. Hejblum et al. [2] pointed out that normalization on such semi-synthetic data would distort some of the true non-DEGs as false-positive DEGs. Because of this, Hejblum et al. [2] found that our results biasedly disfavor the five differential expression (DE) methods (DESeq2, edgeR, limma-voom, dearseq, and NOISeq) except for the Wilcoxon rank-sum test because we applied the five DE methods with their built-in normalization, but we used the Wilcoxon rank-sum test without normalization. Second, Hejblum et al. [2] claimed that dearseq outperformed the other five DE methods. Regarding the first point, we would like to thank Hejblum et al. for pointing out the bias caused by normalization on the semi-synthetic data, an issue we unintentionally omitted because we treated each DE method except for the Wilcoxon rank-sum test as a whole pipeline. Accordingly, here, we re-performed the power comparison analysis under the “no normalization” scheme proposed by Hejblum et al. [2]. Regarding the second point, we have a different conclusion based on our new analysis results. We observed that the Wilcoxon rank-sum test remains the most robust method compared to the other five DE methods in terms of false discovery rate (FDR) control and power performance.

The central point in Yang et al. [3] is that winsorization, a procedure to truncate each gene’s large outlier values beyond a certain empirical percentile (e.g., the 95th percentile) to the percentile, can fix the exaggerated false-positive issue of DESeq2 and edgeR. Yang et al. concluded that outliers caused false-positive exaggeration, and winsorization provided an easy fix. However, Yang et al. conducted an analysis only based on permuted data without true DEGs, and they did not consider semi-synthetic data where true DEGs existed for power evaluation. In this response, we used a model-based strategy to generate semi-synthetic no-outlier RNA-seq data under the negative binomial (NB) model assumption of DESeq2 and edgeR, with true DEGs and non-DEGs. Then, we compared the Wilcoxon rank-sum test with DESeq2 and edgeR under various thresholds of winsorization. Our results show that winsorization cannot fix the inflated FDR issue of DESeq2 and edgeR on our model-based semi-synthetic data, and that the Wilcoxon rank-sum test still performs better in FDR control and power performance. Besides, setting the winsorization threshold is a challenging question in practice. While Yang et al. proposed to set the threshold by controlling the false discoveries made from permuted data and maximizing the discoveries made from real data, this strategy potentially suffers the double-dipping issue (testing a data-driven null hypothesis using the same data) that may hurdle the validity of statistical tests.

In response to the two correspondences, here we added new real data-based simulation analyses to compare the six DE methods. Consistent with our published study [1], we used real RNA-seq data from two conditions to generate semi-synthetic RNA-seq data, which contain ground truths of DEGs and non-DEGs (referred to as true DEGs and true non-DEGs, respectively), for benchmarking the FDR and power of each DE method. To make our benchmark more comprehensive, we employed two simulation strategies to generate semi-synthetic data, including the permutation-based strategy we had used in our published study [1] and a newly added model-based strategy, which (1) satisfies the NB model assumption used in DESeq2 and edgeR and (2) ensures that the semi-synthetic data contain no outliers.

### Two strategies for generating semi-synthetic RNA-seq data from real RNA-seq data

Knowledge of true DEGs and non-DEGs is necessary for evaluating the FDR and power of a DE method. The permuted data that led to the major finding of our published study (Fig. 1 in [1]) only contained true non-DEGs and thus could not be used for power evaluation. Hence, in our published study [1], we also generated semi-synthetic data containing both true DEGs and true non-DEGs. Before the semi-synthetic data generation, we defined the true DEGs as those identified by all six DE methods (DESeq2, edgeR, limma-voom, dearseq, NOISeq, and the Wilcoxon rank-sum test) from the real data at a highly stringent FDR threshold (0.0001%), and we defined the true non-DEGs as the rest of genes. Then, to generate semi-synthetic data from the real data (Fig. 1a, top-middle), we preserved the true DEGs’ unnormalized read counts in the real data, and we independently permuted each true non-DEG’s unnormalized read counts across the real samples. Here, we refer to this previously used generation strategy as the *permutation-based strategy* (Fig. 1a, top-left, later referred to as scheme 2), which has the advantage of preserving the read count values in the real data (Fig. 1a, top-left vs. top-middle).

An alternative strategy to generate semi-synthetic data is the *model-based strategy* (Fig. 1a, top-right), which guarantees that the synthetic data satisfy the NB model assumption required by edgeR and DESeq. This model-based strategy, which we implemented in this response to Yang et al. [3], enabled us to evaluate DESeq2 and edgeR, with or without the winsorization step, under an ideal scenario that the two methods’ model assumption holds. Hence, we can better dissect the reason behind the two methods’ inflated FDR issue. Unlike the permutation-based strategy, this model-based strategy does not preserve the read count values in the real data (Fig. 1a, top-right vs. top-middle). In detail, in the model-based strategy, we first fit a multi-gene distribution (by considering every RNA-seq sample as a draw from the distribution) to the real data (Fig. 1a, top-left) using the model-based simulator scDesign3 [4], which can mimic real RNA-seq data by preserving gene–gene rank correlations and allow us to specify each gene’s distribution. For each true DEG, we specified a per-condition NB distribution fitted to the gene’s real data counts under each condition; for each true non-DEG, we specified one NB distribution fitted by pooling the gene’s real data counts from both conditions. Then, we generated semi-synthetic data by sampling from the fitted multi-gene distribution.

### Response to Hejblum et al. [2]

#### *Key messages in Hejblum *et al*. *[2]

The correspondence by Hejblum et al. [2] has two major points. First, they pointed out that normalization used on the permutation-based semi-synthetic data led to false-positive DEGs, making the FDR comparison results biasedly disfavor the five DE methods (DESeq2, edgeR, limma-voom, dearseq, and NOISeq) that include built-in normalization. The affected results include Fig. 2 and Figures S20-S30 in our published study [1]. Second, dearseq outperforms the Wilcoxon rank-sum test under scheme 3 and otherwise offers competitive performance on par with the other methods.

Regarding the first point, more specifically, when we ran the Wilcoxon rank-sum test on permutation-based semi-synthetic data to verify the FDR control, we did not include a normalization step; on the other hand, when we ran the other five DE methods (DESeq2, edgeR, limma-voom, dearseq, and NOISeq), we had used each method’s built-in normalization. Hence, our comparison results (Fig. 2 and Figures S20-S30 in [1]) did not put the Wilcoxon rank-sum test on the same ground as the other five DE methods in terms of the use of normalization. Hejblum et al. showed that when the Wilcoxon rank-sum test was used after they applied normalization to the semi-synthetic data, it also had an inflated FDR issue as the other five DE methods.

To explain their first point, Hejblum et al. [2] summarized three schemes for using permutation-based semi-synthetic data: (1) “permutation first,” (2) “no normalization,” and (3) “normalization first.” Specifically, scheme 1 “permutation first” means that semi-synthetic data is generated by the permutation-based strategy from real count data, followed by normalization and then DE analysis (Fig. 1a, bottom-left); scheme 2 “no normalization” means that semi-synthetic data is generated by the permutation-based strategy from real count data, followed by DE analysis directly, without normalization (Fig. 1a, top-left); scheme 3 “normalization first” means that semi-synthetic data is generated by the permutation-based strategy from normalized real data, which are no longer counts, followed by DE analysis directly (Fig. 1a, bottom-right).

Our previous analysis had used scheme 1 for DESeq2, edgeR, limma-voom, NOISeq, and dearseq, but scheme 2 for the Wilcoxon rank-sum test (Fig. 2 and Figures S20-S30 in [1]). Unlike schemes 1 and 2, scheme 3 requires a different way of generating semi-synthetic data (Table 1).

**Table 1:** Properties and usages of the three (semi-synthetic data generation and DE method implementation) schemes proposed in Hejblum et al. [2]

Scheme	Unit of gene expression levels in semi-synthetic data	DE method used with normalization?	Compatible with all DE methods?	Usage in our published study Li et al. [1]	Usage in the correspondence Hejblum et al. [2]	Usage in this response
(1) Permutation first (Fig. 1a, bottom-left)	Unnormalized read counts	Yes	Yes	• dearseq (*original package with normalization*) • DESeq2, edgeR, limma-voom, and NOISeq (*unaltered pipelines with normalization*)	• Wilcoxon (*normalization added before the test*) • dearseq (***v1.7.2 and v1.13.1***) *with normalization* • DESeq2, edgeR, limma-voom, and NOISeq (*unaltered pipelines with normalization*)	N/A due to FDR inflation
(2) No normalization (Fig. 1a, top-left)	Unnormalized read counts	No	Yes	Wilcoxon	• Wilcoxon • dearseq (***v1.7.2 and v1.13.1***) *without normalization*^a^ • DESeq2, edgeR, limma-voom, and NOISeq (*unaltered pipelines without normalization*)	• Wilcoxon • dearseq (*updated package v1.8.1 without normalization*) • DESeq2; edgeR; limma-voom, and NOISeq (*unaltered pipelines without normalization*)
(3) Normalization first (Fig. 1a, bottom-right)	Normalized expression levels	No	No	N/A	• Wilcoxon • dearseq (***v1.7.2 and v1.13.1***) *without normalization* • DESeq2, edgeR, limma-voom, and NOISeq (*permutation added after normalization inside each pipeline*)	Discussion of Wilcoxon and dearseq (*updated package v1.14.0 without normalization*) based on the results in Hejblum et al. [2]

^a^The original dearseq package (version 1.6.1, which we used in our published study [1]) did not run with our specific simulated count data without normalization as used in our published study [1]. v1.8.1 runs with this dataset. Here, when we did the new analysis in response to the submitted correspondence of Hejblum et al. [2], we used dearseq (version 1.8.1). Then, the dearseq package was updated to v1.13.1, which was used in the updated correspondence of Hejblum et al. [2]

Regarding the second point raised by Hejblum et al. [2], they re-compared the six DE methods under each of the three schemes and concluded that dearseq [5] outperformed the other five methods, including the Wilcoxon rank-sum test. Our conclusion is different. In the section titled “Our new analysis confirms that the Wilcoxon rank-sum test remains the most robust method under the “no normalization” scheme 2,” we added a new analysis to compare the six DE methods under scheme 2, which we think is the only valid, feasible scheme among the three schemes (explained in the following sections: “We agree with Hejblum et al. that no normalization should be performed if only a portion of genes is permuted to become true non-DEGs,” “Our new simulation analysis confirms that normalization on samples without batch effects and containing true DEGs would inflate false discoveries,” “Our major findings in [1] that DESeq2 and edgeR have exaggerated false discoveries still hold,” “The “normalization first” scheme 3 alters DE method implementation and is thus not-so-realistic,” and “Our rationale of recommending the “no normalization” scheme 2”); our result suggests that the Wilcoxon rank-sum test remains the most robust method among the six DE methods. Additionally, we observed that the Wilcoxon rank-sum test is a robust, top performer in the analysis of Hejblum et al. under their recommended scheme 3 (the section titled “The analysis of Hejblum et al. [2] also suggests that the Wilcoxon rank-sum test is a top performer under scheme 3”), although we regard scheme 3 as a not-so-feasible scheme because it needs to alter DE method implementation by adding permutation after the built-in normalization.

### *We agree with Hejblum *et al*. that no normalization should be performed if only a portion of genes is permuted to become true non-DEGs*

We agree with the first point made by Hejblum et al. that our previous simulation analysis in our published study [1] was unintentionally biased, where the Wilcoxon rank-sum test was run under scheme 2, but the other five DE methods including dearseq were run as whole pipelines (including built-in normalization) under scheme 1.

Hejblum et al. pointed out that post-permutation normalization, i.e., scheme 1, would distort each true non-DEG’s expression levels under the two conditions (because true DEGs’ different expression counts in different samples would make the samples have different library sizes) and would thus cause some true non-DEGs to be identified as false positives. We concur on this point. In fact, we also noticed that scheme 1 would inflate the FDR, and this is why we used scheme 2 for the Wilcoxon rank-sum test, which is not an RNA-seq-specific software package and thus does not include a built-in normalization step. However, the other five RNA-seq-specific DE methods include built-in normalization, and users would likely use them as whole pipelines by not removing the built-in normalization step. While these methods are used as black-box pipelines by most users, it is crucial to be conscientious with their built-in normalization steps when we evaluate the FDR and power of these methods using semi-synthetic data with ground truths, which can be distorted by the normalization steps within these pipelines. Hence, while we agree that running these methods as pipelines, i.e., under scheme 1, was unfair for evaluating the FDR control of their statistical tests, we believe that our previous results (Fig. 2 and Figures S20-S30 in [1]), in conjunction with Hejblum et al.’s results, are meaningful for showing the risks of using bioinformatics tools as black-box pipelines.

While unfair, our previous analysis was not intentionally biased against dearseq. The reason was that the dearseq R package we used in our published study [1] (v1.6.1, Bioconductor date October 26, 2021) did not support the use of our simulated count data without normalization. Hence, we were unable to assess dearseq under scheme 2 like the Wilcoxon rank-sum test. The detail is in Additional file 1, an R Markdown file recording how dearseq was used in our published study. Note that the dearseq R package was updated after our publication [1] to allow scheme 2 (see Table 1). Hence, we used dearseq v1.8.1 (the most up-to-date version at the time of our new analysis) in this response.

### *Our new simulation analysis confirms that normalization on samples without batch effects and containing true DEGs would inflate false discoveries*

In our opinion, the fundamental reason behind the first point raised by Hejblum et al. is that no normalization should be performed on samples without batch effects and containing true DEGs, the case for the permutation-based semi-synthetic data, where true DEGs and true non-DEGs are defined by assuming no batch effects.

To demonstrate that bias normalization can be introduced on samples without batch effects and containing true DEGs, we simulated samples of two conditions, in which each true non-DEG has counts from the same NB distribution under both conditions, and each true DEG has different NB distributions for the two conditions. On these simulated samples without batch effects, if we first applied the trimmed mean of *M*-values (TMM) normalization [6] and then the Wilcoxon rank-sum test for DEG identification, then the actual FDR was 7.9% at a target FDR threshold of 5%. In contrast, if we directly applied the Wilcoxon rank-sum test without normalization, then the actual FDR was 3.5% at the same target FDR threshold of 5%. This result suggests that, in the absence of batch effects, normalization would introduce bias and should not be used.

The bias induced by unnecessary normalization is also demonstrated in Fig. 1a. There is one true DEG and one true non-DEG in the permutation-based semi-synthetic data (Fig. 1a, top-left). However, the non-DEG exhibits an obvious difference in the mean expression levels between the two conditions after the normalization, making it likely to be identified as a false-positive DEG (Fig. 1a, bottom-left).

In our published study [1], we generated semi-synthetic data using the permutation-based strategy. For the true DEGs, we fixed their unnormalized read counts as in the real data’s two conditions. For the remaining genes as true non-DEGs, we randomly permuted each gene’s unnormalized read counts between the real data’s two conditions. Given that we did not assume batch effects to exist in the semi-synthetic data when we defined true DEGs and true non-DEGs, normalization is unnecessary and would only serve to skew the defined ground truth. Hence, we conclude that no normalization should be performed on the permutation-based semi-synthetic data (i.e., scheme 2 in Table 1).

### *Our major findings in *[1]* that DESeq2 and edgeR have exaggerated false discoveries still hold*

It is worth noting that permutation remains a valid sanity check of DE methods (with or without built-in normalization) when all genes are permutated. In such permuted data, all genes are true non-DEGs, and all samples are exchangeable (Fig. 1b, left). Hence, a normalization procedure, if appropriate, should not introduce biases to exchangeable samples and distort the ground truth (Fig. 1b, right). As our major point about exaggerated false discoveries (Fig. 1 in our published study [1]) was based on such permuted data, our observation that DESeq2 and edgeR had exaggerated false discoveries when analyzing human population samples remains valid. However, as no true DEGs existed in such permuted data, we could only use these data to evaluate the FDR of a DE method, but not the power. This motivated us to generate semi-synthetic data that contained both true DEGs and non-DEGs (see the section titled “Two strategies for generating semi-synthetic RNA-seq data from real RNA-seq data”).

### *The “normalization first” scheme 3 alters DE method implementation and is thus not-so-realistic*

Realizing the issue with scheme 1, Hejblum et al. proposed scheme 3 (Table 1) to generate semi-synthetic data in a different, normalization-then-permutation way: first normalizing real data within each condition; then generating semi-synthetic data by fixing true DEGs’ normalized expression levels in the two conditions and randomly permuting each true non-DEG’s normalized expression levels between the two conditions. It is true that scheme 3, like the “no normalization” scheme 2, does not perform the post-permutation normalization, so true non-DEGs’ expression levels would not be distorted and then become false positives. However, we argue that scheme 3 would generate semi-synthetic data containing normalized expression levels that are no longer counts, making many DE methods that require count data as input become inapplicable. In fact, Hejblum et al. [2] had to alter each DE method by adding a permutation step after the normalization step, making scheme 3 not only a semi-synthetic data generation scheme but also an altered implementation of DE methods.

### *Our rationale of recommending the “no normalization” scheme 2*

Given the fact that scheme 1 causes FDR inflation and scheme 3 must alter DE method implementation, our recommendation is the “no normalization” scheme 2. Moreover, there are different normalization methods available, and the choice of normalization methods remains an open question [7–9]. Using scheme 2 allows us to concentrate on the performance of DE methods without the confounding effect of the normalization method choice.

It is important to emphasize that all three schemes apply only to FDR and power evaluation of DE methods using permutation-based semi-synthetic data, not real data analysis. For real data, unless analysts are confident about the absence of batch effects, we recommend the use of normalization prior to conducting DE analysis.

### *Our new analysis confirms that the Wilcoxon rank-sum test remains the most robust method under the “no normalization” scheme 2*

**Fig. 3:**
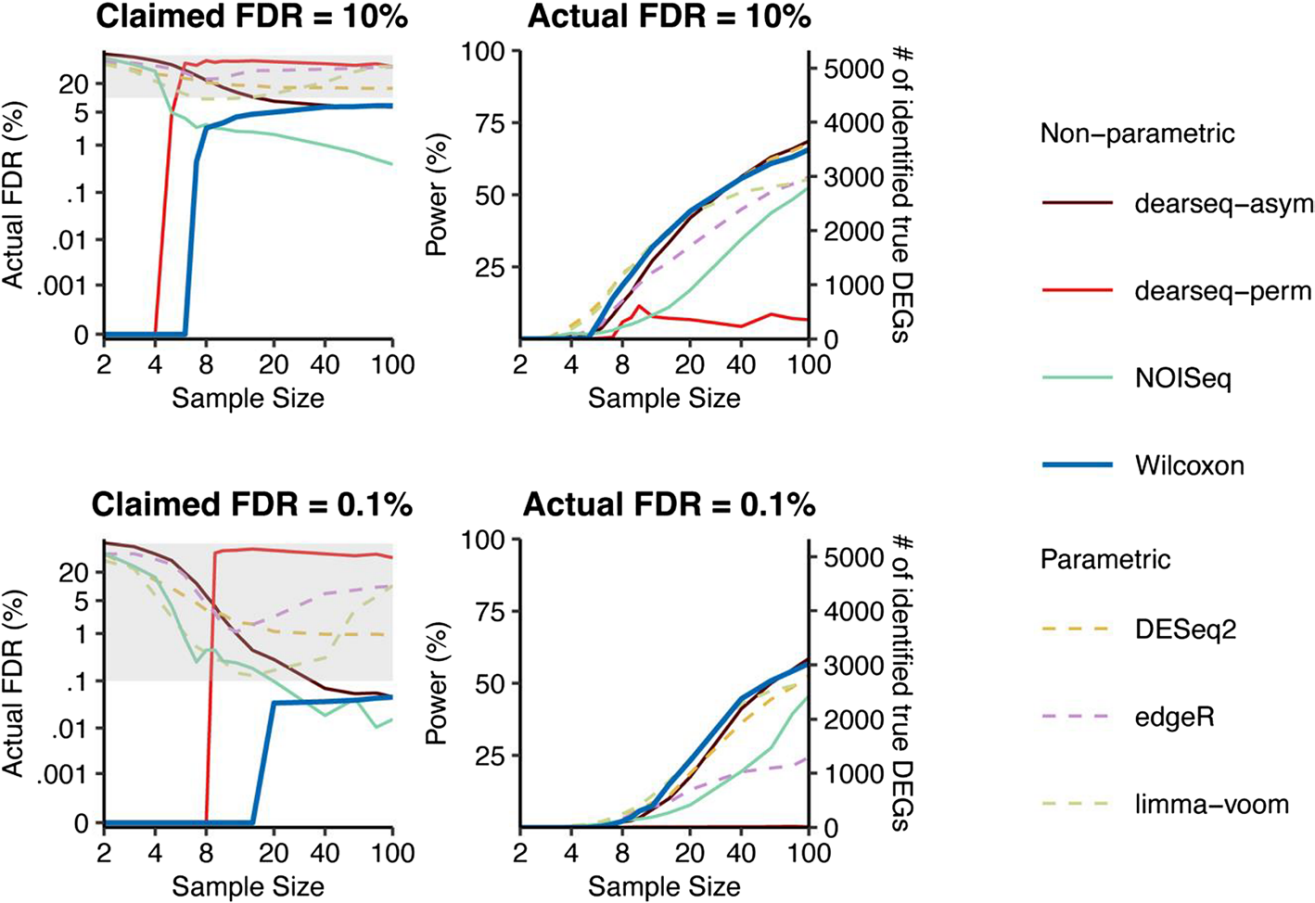
Comparison of DE methods on semi-synthetic data (with varying sample sizes) generated using the permutation-based strategy from GTEx data of heart left ventricle vs. atrial appendage under the data generation scheme 2. The FDR control (left panel) and power given the actual FDRs (right panel) for a range of per-condition sample sizes from 2 to 100, under FDR thresholds (i.e., claimed FDRs) of 10% (top panel) and 0.1% (bottom panel). The claimed FDRs, actual FDRs, and power were all calculated as the averages of 50 semi-synthetic datasets with the same per-condition sample size

Regarding the second major point of Hejblum et al. [2] that dearseq outperforms the Wilcoxon rank-sum test under scheme 3 and otherwise offers competitive performance on par with the other methods, we performed a new analysis for FDR and power evaluation under our recommended "no normalization" scheme 2 for the six DE methods, including the updated dearseq (v1.8.1) package that allows this scheme and was released after our published study [1]. Our updated results show that the Wilcoxon rank-sum test remains the most robust method compared to the other five DE methods (Figs. 2 and 3).

Importantly, dearseq has two versions: dearseq-perm uses a permutation test for *p*-value calculation, and dearseq-asym uses an asymptotic null distribution for *p*-value calculation.

In this response, under scheme 2, we first re-ran the six DE methods on the permutation-based semi-synthetic data generated in our published study [1] and obtained results similar to those in Hejblum et al. [2]. As shown in Figs. 2 and 3, dearseq-perm cannot control the FDR. The other version, dearseq-asym, also cannot control the FDR when the sample sizes are 20 vs. 20 (Fig. 2), and it controls the FDR only when the sample size is large enough (e.g., sample size ~ 40 when the target FDR is 0.1%; Fig. 3). In contrast, the Wilcoxon rank-sum test has consistent FDR control across all sample sizes. Figures 2 and 3 also show that under the same actual FDR, dearseq-perm has the worst power among all DE methods, while dearseq-asym has no obvious power advantage over the Wilcoxon rank-sum test. Another drawback of dearseq-perm is its long computational time. In our benchmark, the average computational time of dearseq-perm under its default setting was 3.5 h on one semi-synthetic dataset (with sample sizes 372 and 386); in contrast, the Wilcoxon rank-sum test spent only 1.5 min on the same dataset, and dearseq-asym spent 1 min. Based on these results, the conclusion in our published study still holds that the Wilcoxon rank-sum test is more robust than dearseq-asym regarding the sample size and outperforms dearseq-perm regarding the FDR control consistency and the statistical power.

### *The analysis of Hejblum *et al*. *[2]* also suggests that the Wilcoxon rank-sum test is a top performer under scheme 3*

Since scheme 3 is not directly applicable to DESeq2 [10], edgeR [11], and limma-voom [12], which only accept gene expression read counts as input data, we chose not to alter their pipelines and then run them under this scheme. Hence, we used the results in Hejblum et al. [2] to compare the Wilcoxon rank-sum test with dearseq-perm and dearseq-asym. Hejblum et al.’s Figs. 1 and 2 showed that dearseq-perm can control the FDR but lacks power, and dearseq-asym can control the FDR only when the sample size is large enough (at least 80 samples per condition). In contrast, the Wilcoxon rank-sum test has consistent FDR control across all sample sizes. Moreover, dearseq-asym has no obvious power advantage over the Wilcoxon rank-sum test. Hence, we conclude that the Wilcoxon rank-sum test is still a good choice under scheme 3, although we do not recommend scheme 3 for the reasons stated in the section titled “The “normalization first” scheme 3 alters DE method implementation and is thus not-so-realistic.”

In summary, after fixing the normalization bias in our published analysis (Fig. 2 in [1]), we claim that our previous conclusion remains: the Wilcoxon rank-sum test is the most robust among the six methods. In particular, the Wilcoxon rank-sum test is advantageous for being fast and able to strictly control the FDR for all sample sizes. In contrast, dearseq-perm is too slow thus impractical, and dearseq-asym cannot control the FDR when the sample size is less than 40 per condition. Nevertheless, we acknowledge dearseq’s advantage for allowing more complex experimental designs, which the Wilcoxon rank-sum test cannot handle but needs extensions such as the probabilistic index models [13].

### Response to Yang et al. [3]

#### *Key messages in Yang *et al*. *[3]

The correspondence by Yang et al. pointed out that edgeR might still be suitable for DE analysis of large-sample RNA-seq datasets after outlier samples are removed by winsorization. To perform winsorization, Yang et al. initially normalized the count data by sample, using the samples’ size factors calculated by the estimateSizeFactors() function in the R package DESeq2. Next, for each gene, the normalized counts that exceeded a certain percentile were truncated to the percentile value. These modified counts were next rescaled by multiplication with the original size factors and then rounded to the nearest integer, yielding the winsorized counts. Finally, the winsorized counts were fed into DESeq2 and edgeR for DEG identification.

After applying the above winsorization procedure, Yang et al. found that edgeR had similar power as the Wilcoxon rank-sum test after the 93rd percentile winsorization was used, and edgeR could control the FDR close to the target level, while DESeq2 still had the FDR inflation issue (Fig. 2B in Yang et al. [3]). Hence, Yang et al. claimed that edgeR could still be used for DE analysis on large-sample RNA-Seq data after winsorization.

However, we assert that a practical challenge is setting the winsorization threshold, a tuning parameter that needs user specification and determines DE analysis results. Yang et al. recommended users permute the datasets many times and select the winsorization threshold as the percentile that controls the false discoveries made from permuted data and maximizes the discoveries made from real data. However, this approach requires running edgeR separately for each threshold on each permutated dataset and is thus computationally intensive. Besides, this strategy suffers the double-dipping issue (selecting the winsorization threshold and performing statistical tests on the same data) that hurdles the validity of statistical tests.

#### The Wilcoxon rank-sum test outperforms DESeq2 and edgeR in the absence of outliers

**Fig. 4:**
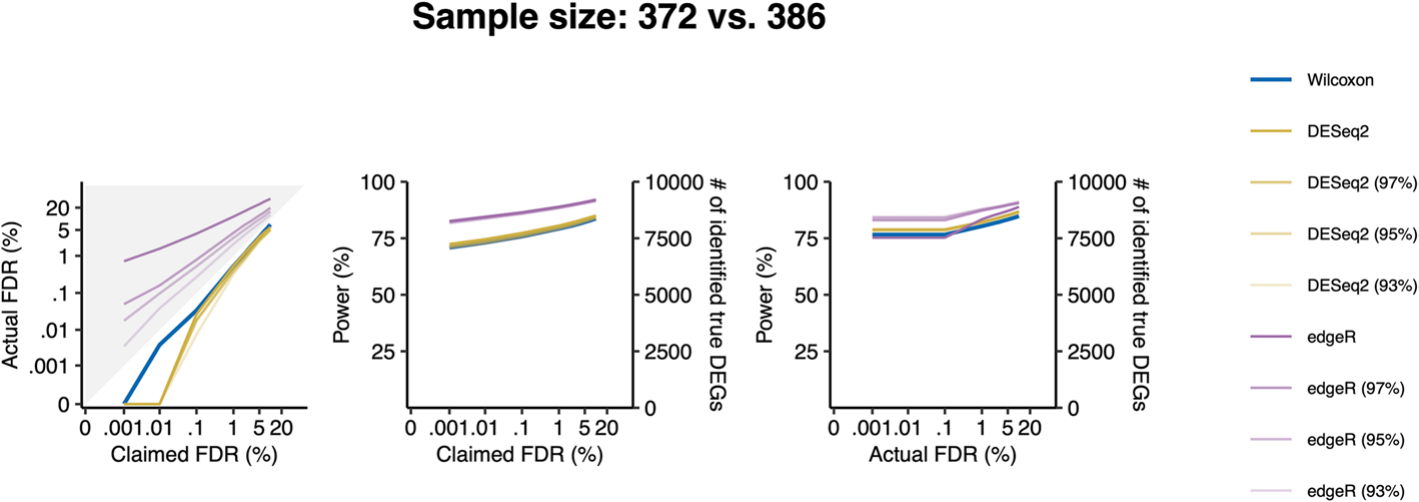
Comparison of DESeq2 without or with winsorization, edgeR without or with winsorization, and Wilcoxon rank-sum test on semi-synthetic data generated using the model-based strategy from GTEx data of heart left ventricle vs. atrial appendage. The FDR control (left panel), power given the claimed FDRs (middle panel), and power given the actual FDRs (right panel) under a range of FDR thresholds (i.e., claimed FDRs) from 0.001 to 5%. DESeq2 and edgeR were applied in default (without winsorization) or with three winsorization thresholds (the 93%, 95%, and 97% quantiles) based on the winsorization procedure in Yang et al. [3] . As the true DEGs and non-DEGs were defined without assuming batch effects, DESeq2 and edgeR were applied without normalization. The claimed FDRs, actual FDRs, and power were all calculated as the averages of 30 semi-semisynthetic datasets generated independently

In this response, we independently investigated whether DESeq2 or edgeR has advantages over the Wilcoxon rank-sum test for large sample-size data in the absence of outliers (because the purpose of winsorization is to remove outliers). We considered an ideal scenario by employing the model-based strategy to generate semi-synthetic data, which contained true DEGs and no outliers, from a real GTEx dataset of heart left ventricle vs. atrial appendage (with sample sizes 376 and 386). Specifically, we set each gene’s marginal distributions under each condition as a NB distribution, satisfying the model assumption of DESeq2 and edgeR. Then, we applied DESeq2 and edgeR in the default mode (no winsorization) or with three winsorization thresholds (the 93%, 95%, and 97% percentiles) to the semi-synthetic data. Our results show that all implementations of DESeq2 and edgeR (the default and the three winsorized variants) did not control the FDRs under the target levels (Fig. 4).

Generated by the model-based strategy, the semi-synthetic data contain no outliers because the model satisfies the assumption of DESeq2 and edgeR. However, we still observed inflated FDRs of DESeq2 and edgeR, suggesting that the existence of outliers is not the only reason for the inflated FDR issue. Another reason may be associated with the inaccurate estimation of the dispersion parameter of each NB distribution, one per gene per condition. This dispersion parameter is a nuisance parameter that, although not of interest, must be estimated before a hypothesis test can be performed on the mean parameter, the parameter of interest. (Note that in DESeq2 and edgeR, the null hypothesis for each gene is that the gene has the same mean parameter, with samples’ size factors adjusted, under the two conditions.) Hence, if the dispersion parameter has an estimation bias, it would likely lead to a mis-calibrated *p*-value for the hypothesis test of the mean parameter. This dispersion parameter estimation issue, if existent, cannot be fixed by winsorization. Indeed, we observed that DESeq2 and edgeR still cannot control the FDR on the model-based semi-synthetic data after winsorization. Hence, we conclude that the Wilcoxon rank sum test still outperforms DESeq2 and edgeR on large sample size data, even allowing for winsorization.

In summary, although DESeq2 and edgeR have fewer false positives after winsorization, (Fig. 4), they still cannot control the FDR on model-based semi-synthetic data that satisfy their model assumption. Moreover, winsorization adds subjectivity to data analysis, and winsorization threshold choice is challenging. Hence, we hold our recommendation in the original study [1] that the Wilcoxon rank-sum test is a preferred and robust choice for large sample-size RNA-seq data if complex experimental design is not involved.

### Final note

We would like to acknowledge the insightful points raised by the two correspondences, which have helped us refine our analysis and strengthen the rigor of our conclusions. We firmly believe that these discussions contribute substantially to the field’s understanding of DE analysis—a topic that may have previously been regarded as a solved issue.

After discussion and analysis in this response, we recommend no normalization in simulation schemes where no batch effects are assumed in defining true DEGs and non-DEGs. Based on our new analysis results in this response, the conclusions in our original study [1] continue to hold. The non-parametric Wilcoxon rank-sum test can consistently control the FDR and achieve good statistical power for large sample-size RNA-seq data. While winsorization that removes outliers may reduce the inflated FDR issue of the parametric DE methods DESeq2 and edgeR, selecting the threshold for winsorization is arbitrary or computationally intensive. This subjectivity issue is also prevalent in other data preprocessing steps, such as the removal of top principal components (PCs) following data transformation, where the number of PCs to remove is subjectively determined. In contrast, the Wilcoxon rank-sum test is a robust approach independent of parameter specifications or arbitrary thresholds.

We would also like to clarify that our published study [1] was in no way a comprehensive benchmark. Many DE methods have been developed in the last decade (including more than 20 methods benchmarked in previous studies; see Table S1 in [1]). It is possible that some methods may outperform the Wilcoxon rank-sum test on specific datasets or under specific settings. Instead of providing the “best” method, an impossible mission given the vast diversity of datasets, our study [1] aimed to emphasize the importance of sanity checks and voice the cautionary message that using popular methods without checks might lead to excessive false positives.

## Supplementary Information

**Supplementary Material 1.**

## Data Availability

All the code and data used to generate the new results can be found in "Data and codes_response.zip" available at Zenodo: https://zenodo.org/records/10948467.
